# TRY-5 Is a Sperm-Activating Protease in *Caenorhabditis elegans* Seminal Fluid

**DOI:** 10.1371/journal.pgen.1002375

**Published:** 2011-11-17

**Authors:** Joseph R. Smith, Gillian M. Stanfield

**Affiliations:** Department of Human Genetics, University of Utah, Salt Lake City, Utah, United States of America; Harvard University, United States of America

## Abstract

Seminal fluid proteins have been shown to play important roles in male reproductive success, but the mechanisms for this regulation remain largely unknown. In *Caenorhabditis elegans*, sperm differentiate from immature spermatids into mature, motile spermatozoa during a process termed sperm activation. For *C. elegans* males, sperm activation occurs during insemination of the hermaphrodite and is thought to be mediated by seminal fluid, but the molecular nature of this activity has not been previously identified. Here we show that TRY-5 is a seminal fluid protease that is required in *C. elegans* for male-mediated sperm activation. We observed that TRY-5::GFP is expressed in the male somatic gonad and is transferred along with sperm to hermaphrodites during mating. In the absence of TRY-5, male seminal fluid loses its potency to transactivate hermaphrodite sperm. However, TRY-5 is not required for either hermaphrodite or male fertility, suggesting that hermaphrodite sperm are normally activated by a distinct hermaphrodite-specific activator to which male sperm are also competent to respond. Within males, TRY-5::GFP localization within the seminal vesicle is antagonized by the protease inhibitor SWM-1. Together, these data suggest that TRY-5 functions as an extracellular activator of *C. elegans* sperm. The presence of TRY-5 within the seminal fluid couples the timing of sperm activation to that of transfer of sperm into the hermaphrodite uterus, where motility must be rapidly acquired. Our results provide insight into how *C. elegans* has adopted sex-specific regulation of sperm motility to accommodate its male-hermaphrodite mode of reproduction.

## Author Summary

Sexual reproduction requires the generation of highly specialized gametes, eggs and sperm, that must encounter one another and fuse together to form a zygote. Males provide not only sperm but also seminal fluid, which contains a variety of factors that promote male fertility through effects on sperm and on female physiology. We have identified a *C. elegans* seminal fluid protease, TRY-5, that regulates sperm activation, the process by which immature spermatids complete their differentiation to a motile form capable of fertilizing an oocyte. We observed release of TRY-5 that coincided with transfer of sperm, coupling the onset of sperm motility to transfer during mating. Although TRY-5 functions only in males, both male and hermaphrodite sperm are capable of responding to it. TRY-5 is not required for fertility, and we propose that a hermaphrodite activator compensates in its absence. Our results reveal how sperm development can be differentially modulated by males and hermaphrodites to promote fertility in each sex, and we identify a novel function for a seminal fluid protein.

## Introduction

A general feature of sexual reproduction is the generation of motile sperm that can navigate to an egg. To assist this process, males transfer their sperm along with seminal fluid, which enhances their reproductive success in a variety of ways (reviewed in [1], [2]). Seminal fluid factors promote sperm survival, motility and fertilizing ability both by directly interacting with sperm and by interacting with tissues of the female to make her reproductive tract a more permissive environment. These factors include seminal fluid-specific proteins, a variety of hormones, and energy sources [2]. In mammals, roles for seminal fluid factors include the regulation of sperm motility and capacitation and the modulation of immune function [2], [3]. Extensive analysis in *Drosophila* has identified many seminal fluid proteins and uncovered roles for several of these factors in sperm storage, sperm competition, female reproductive behavior and physiology, and other processes [4]. Due to their potential for influencing reproductive success, components of seminal fluid represent a forum for both conflict and cooperation between the sexes [1], [5].

The androdioecious nematode *Caenorhabditis elegans* provides an opportunity to analyze sperm development and function in a context where both sexes produce sperm and can differentially regulate gamete function to promote their fertility. Hermaphrodites are self-fertilizing; during development, they produce a store of “self” sperm, which can be used to fertilize their eggs. Males mate with and transfer sperm to hermaphrodites. Males are not required for reproduction to occur, and in their absence self sperm are used with extremely high efficiency; more than 99% of self sperm are used. However, if male sperm are present, then they preferentially fertilize eggs [6].

*C. elegans* sperm, like those of other nematodes, lack flagella; instead, they move by crawling using a pseudopod [6]–[9]. Motility is acquired during sperm activation, a process analogous to spermiogenesis in flagellate sperm, in which haploid spermatids undergo a dramatic cellular rearrangement to become competent for both directional motility and fertilization of an oocyte [6]. While most aspects of sperm development are similar in males and hermaphrodites, the timing and context of activation differ in the two sexes. In hermaphrodites, spermatids activate when they move into the spermathecae, regions of the gonad where sperm are stored and fertilization occurs. In males, sperm are stored in a non-activated form and become activated after mating and transfer to a hermaphrodite ([6] and unpublished observations). Sperm also can be activated *in vitro* in response to treatment with a variety of factors, including an ionophore (monensin), proteases (Pronase), a weak base (triethanolamine/TEA), and an ion channel inhibitor (4,4′-diisothiocyano-2,2′-stilbenedisulfonic acid/DIDS) [10]–[13]. This ability, together with the observation that sperm generally activate *in vivo* in response to a change in location, suggests that activation is controlled by extracellular signals.

Genes that regulate sperm activation show distinct requirements in hermaphrodites and males. The activity of a set of five genes termed the “*spe-8* group” (*spe-8*, *-12*, *-19*, *-27*, and *-29*) is required specifically for hermaphrodites to activate their self sperm; hermaphrodites mutant for any one of these genes are self sterile, while mutant males are fertile [12], [14]–[19]. Mating of *spe-8* group mutant hermaphrodites with males results in self-sperm activation (“transactivation”) and can restore self fertility, suggesting that males provide their own activator to which *spe-8* group hermaphrodite sperm can respond [12]. *spe-8* group functions are dispensable for production of this activator, since both wild-type and *spe-8* group males are competent for transactivating hermaphrodite sperm. While these analyses indicate that there are differences in the intracellular pathways by which sperm are activated in the two sexes, the functions of individual activation genes are not strictly limited to a specific sex. *spe-8* group mutant male sperm show some defects, failing to activate in response to Pronase *in vitro* [12], [15], [17], [19]. Furthermore, some *spe-8* group activity is likely required for sperm to transactivate, since animals harboring *spe-8* group null alleles appear to be insensitive to male activator [17], [19]. While most analysis has focused on hermaphrodite sperm activation, a gene with a male-biased effect has been identified as well. Activity of an extracellular trypsin inhibitor-like protein, SWM-1, is required in males to prevent premature activation from occurring prior to mating, and *swm-1* mutant males are infertile due to failure to transfer activated sperm [20]. *swm-1* activity is dispensable in hermaphrodites, though loss of *swm-1* improves fertility in a sensitized *spe-8* group mutant background [20]. The finding that a protease inhibitor regulates activation in males, combined with the ability of proteases to activate sperm *in vitro*, suggested that protease activity could signal activation *in vivo*. However, the endogenous activator has not been identified as yet in either sex.

Here, we report the identification of a trypsin-family serine protease, TRY-5, which has the properties expected of a male sperm activator. Loss of *try-5* suppresses mutations in *swm-1*. Furthermore, during mating, TRY-5 is released from the somatic gonad and transferred along with sperm, thus coupling the onset of sperm motility to the time of their transfer to a hermaphrodite. Within the male gonad, TRY-5 activity must be held in check to ensure male fertility. Strikingly, TRY-5 is not required for male fertility, but strains lacking both *try-5* and *spe-8* group activation functions are totally sterile, confirming that while male and hermaphrodite sperm motility is induced by distinct signals, the two pathways are redundant. In summary, TRY-5 is the first factor demonstrated to be a transferred component of seminal fluid in *C. elegans*, where it plays a key role in male-specific regulation of sperm function.

## Results

### *C. elegans* male sperm activation is regulated by a protease

**Figure 1:**
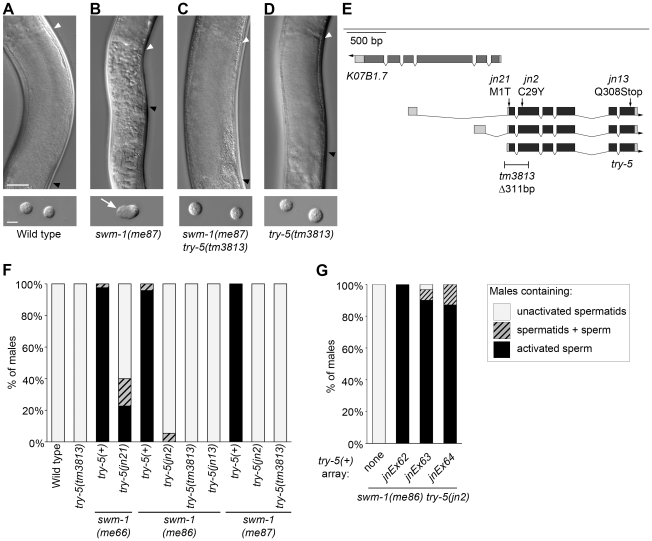
Mutations in the serine protease gene *try-5* suppress premature sperm activation in *swm-1* mutant males. Sperm activation was examined in staged 48 hr post-L4 adult virgin males. (A–D) Differential interference contrast (DIC) images showing sperm morphology in the indicated strains. Top row: Images of intact males in the region of the seminal vesicle (SV), where sperm are stored. Strains shown in A, C and D have round spermatids, which pack together in the SV of intact animals to form a uniformly grainy appearance. The strain shown in B contains activated sperm, resulting in a rough appearance. Arrowheads indicate the anterior (white) and posterior (black) boundaries of the SV. Scale bar, 25 µm. Bottom row: DIC images of dissected sperm. Arrow indicates a pseudopod. Scale bar, 5 µm. (See Figure S1 for high-resolution versions of the images in B, C.) (E) Schematic of the *try-5* region. We used RACE and RT-PCR to characterize *try-5* transcripts, generating updated gene models as compared to the WormBase prediction ([59]; accession numbers JN651275, JN651276 and JN651277). On gene models, darker shading indicates predicted coding regions and arrow indicates the direction of transcription. The positions of mutations in *try-5* are indicated along with their predicted effects. (See also Figure S2.) (F) Quantitation of suppression of *swm-1* by mutations in *try-5*. Stacked columns indicate the percent of males containing either only activated sperm (black), a mixture of spermatids and activated sperm (hatched), or only non-activated spermatids (grey). At least 30 animals were scored for each genotype. (G) A *try-5* transgene restores sperm activation in *swm-1* mutant males. Sperm activation was assayed in *unc-119; swm-1(me86) try-5(jn2) him-5* males bearing extrachromosomal arrays of pJRS14, which contains *try-5*(+) and *C. briggsae unc-119*(+) (Tables S1 and S2). Data from three independent transgenic lines are shown. Key for stacked columns as in (F). Between 22 and 31 animals were scored for each genotype.

In wild-type males, sperm are stored in the inactive form within the seminal vesicle (Figure 1A) and become activated after transfer to a hermaphrodite ([6] and unpublished data). Mutations in the secreted protease inhibitor SWM-1 result in premature sperm activation within males (Figure 1B, 1A, [20]). We predicted that loss of activation-promoting factors should suppress this phenotype. To identify such factors, we performed genetic screens for suppressors of premature sperm activation caused by the partial loss-of-function alleles *swm-1(me86)* or *swm-1(me66)* (G.M.S., unpublished; [20]). Among the *swm-1* suppressor mutants, we identified three alleles of the serine protease gene *try-5* (Figure 1E and 1F). We subsequently obtained *tm3813*, a deletion affecting the 5′ end of the *try-5* coding region (gift of S. Mitani, National Bioresource Project, Japan), and showed that it also suppressed *swm-1(me86)* (Figure 1F). Suppression of the premature activation phenotype in *swm-1 try-5* double mutants was rescued by a genomic fragment containing the full-length *try-5* gene (Figure 1G, Tables S1, S2, S4 and data not shown), confirming that *try-5* was responsible for this effect.

In parallel to our forward genetic screen, we also tested individual serine proteases for a role in sperm activation. We used RNA interference to reduce the function of individual protease genes in a *swm-1* mutant background and screened for effects on premature activation in males. Among the tested proteases, only reduction of *try-5* resulted in strong suppression (Materials and Methods and data not shown), consistent with our finding that *try-5* is a regulator of sperm activation.

Based on conservation of its sequence and domain structure [21] with those of the trypsin-like superfamily, *try-5* is predicted to encode a trypsin-class serine protease. This family of proteases contains numerous members in eukaryotes and regulates many processes, including blood coagulation, developmental signaling and fertilization [22]. Specific residues that form the protease active site are conserved in TRY-5, and the presence of a signal sequence on the N terminus of the protein suggests that it is secreted (Figure S2). While TRY-5 has clear orthologs in other closely related nematodes, it is divergent from serine proteases in more distantly related species (data not shown). In addition, its substrate-binding region is divergent from those of trypsin family members with characterized substrate specificities [23].

We initially identified *try-5* using partial loss-of-function alleles of *swm-1*. To determine whether mutations in *try-*5 are capable of suppressing a *swm-1* null, we examined animals harboring both the null allele *swm-1(me87)* and an allele of *try-5*. We found that whereas *swm-1(me87)* mutant males contain activated sperm [20], *swm-1(me87) try-5(jn2)* and *swm-1(me87) try-5(tm3813)* males contained non-activated sperm like those found in the wild type or in a *try-5* mutant ([20], Figure 1A–1D and 1F, Figure S1). In summary, these results indicate that the protease TRY-5 is responsible for the premature sperm activation and associated loss of fertility that occur in *swm-1* mutant males and suggest that the function of SWM-1 is to inhibit TRY-5 activity within the male.

### Male sperm can activate in the absence of *try-5*

**Figure 2:**
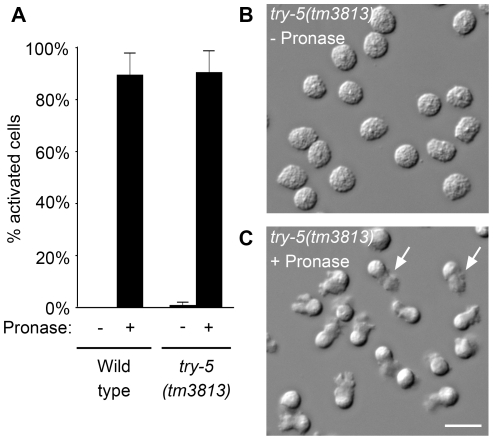
*try-5* mutant sperm are capable of activation. Sperm were assayed for activation in response to protease treatment. Wild-type or *try-5(tm3813)* mutant sperm were dissected and incubated in 200 µg/mL Pronase for 25 min; activation was scored every 5 min based on the presence or absence of a pseudopod. (A) Maximal activation observed at a single time point during the assay; average of 3 repeats. Error bars represent standard error of the mean. (B,C) DIC images of *try-5(tm3813)* sperm prior to Pronase treatment (B) and the same field of cells at 20 min (C). See also Video S1. Arrows indicate the pseudopodia for two of the activated cells. Scale bar, 10 µm.

To see if *try-5* is required for male sperm to activate, we assessed the ability of *try-5* mutant sperm to respond to treatments that bypass normal activation signals. Wild-type sperm can be activated *in vitro* by treatment with any of a variety of compounds [10]–[13]. Since TRY-5 is predicted to be a protease, we first assayed the ability of *try-5* mutant spermatids to activate in response to Pronase treatment. In the absence of Pronase, both wild-type and *try-5* mutant sperm remained non-activated (Figure 2A and 2B). Within 5 to 10 min after addition of Pronase, the majority of sperm cells developed a pseudopod, consistent with activation (Figure 2A and 2C, Video S1). These cells were capable of motility, as they were observed crawling across the microscope slide (note altered positions of some cells in Figure 2B versus Figure 2C). There was no significant difference in either the level of activation (Figure 2A; P = 0.89, Student's t test) or the rate of activation (data not shown) of *try-5* mutant sperm as compared to the wild type. We then tested the ability of *try-5(tm3813)* spermatids to activate in response to treatment with a second known activator, the weak base TEA. When treated with TEA, *try-5* mutant spermatids activated at levels similar to wild-type sperm (data not shown). Thus, *try-5* is not required for sperm activation initiated *in vitro* either by exogenous proteases or TEA. This result distinguishes *try-5* mutants from the previously-characterized *spe-8* group mutants, for which sperm activate normally when treated with TEA, but arrest at a partially-activated, “spiky” stage in response to Pronase [12], [15], [17], [19].

We next determined if *try-5* is required for activation induced by loss of the intracellular activation inhibitor *spe-6*. SPE-6 is a sperm casein kinase 1-like protein that functions at two points during spermatogenesis: during spermatogenic cell divisions [24] and later during sperm activation [18]. Specific mutations in *spe-6* allow spermatogenesis to occur but lead to premature sperm activation in males, a phenotype that is thought to be independent of extracellular signaling [18]. To determine whether *try-5* function is required for the premature sperm activation phenotype of *spe-6*, we assayed sperm activation in *spe-6(hc163); try-5(tm3813)* and *spe-6(hc163); try-5(jn2)* mutant males. We found that, like *spe-6(hc163)* mutant males, *spe-6(hc163); try-5* males contained activated sperm (Table 1) and their appearance was indistinguishable from that of the *spe-6* mutant (data not shown). Thus, TRY-5 activity does not function downstream of the sperm protein SPE-6. Together, the ability of *try-5* sperm to activate in response to either *in vitro* activators or loss of an intracellular inhibitor indicates that TRY-5 is not required for the subcellular rearrangements of sperm activation. Rather, these data suggest a regulatory role for this protease in signaling sperm to initiate the activation process.

**Table 1:** TRY-5 is not required for activation in *spe-6* animals.

Genotype^1^	% Act^2^	n
wild type	97	32
*spe-6(hc163)*	100	40
*try-5(jn2)*	0	43
*try-5(tm3813)*	0	53
*spe-6(hc163); try-5(jn2)*	100	42
*spe-6(hc163); try-5(tm3813)*	100	61

1 All strains also contained the mutation *dpy-18(e364)*.

2 Percent of 48 hr post-L4 males containing activated sperm.

### *try-5* is not required for fertility

**Figure 3:**
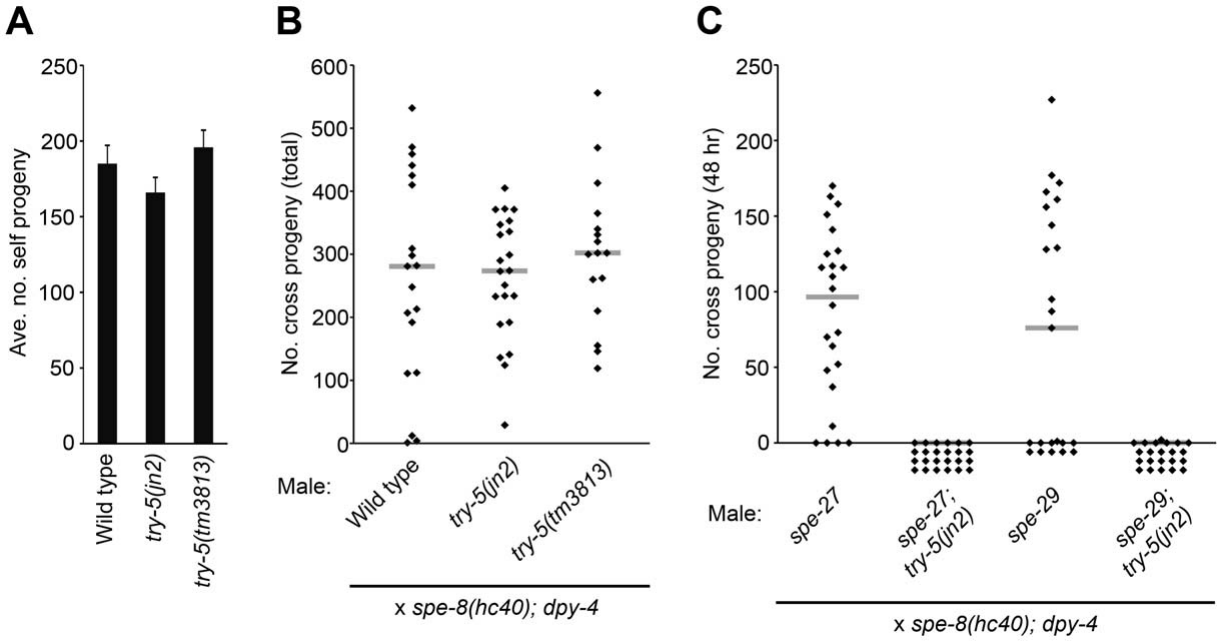
*try-5* is not required for fertility and functions in parallel to the *spe-8* group. Hermaphrodite and/or male fertility was measured for *try-5* mutant strains. (A) *try-5* hermaphrodites show normal fertility. Columns indicate average brood size of self-fertilizing hermaphrodites. Error bars represent standard error of the mean. (B) *try-5* males show normal fertility. Males were placed with *spe-8(hc40); dpy-4* hermaphrodites in 1∶1 crosses for 48 hr and the entire brood size was measured by counting the total number of non-Dumpy cross progeny. *try-5* mutants were not significantly different from the control (*try-5(jn2)*, p = 0.95; *try-5(tm3813)*, p = 0.40; Mann-Whitney U Test). (C) *try-5* is required for fertility in *spe-8* group mutant males. Males were placed with *spe-8(hc40); dpy-4* hermaphrodites in 1∶1 crosses for 48 hr and the number of non-Dumpy cross progeny produced during the mating period was counted. Infertility of *spe-8* group; *try-5* males was due to failure of sperm to activate or migrate (Table S3). (B,C) Each point represents the result of an individual cross; gray lines represent medians. Sets of crosses with each genotype were repeated at least twice and a representative set of data is shown. (See also Figure S3.)

Since activation is necessary to generate mature, motile spermatozoa that are competent for fertilization, failure to activate results in infertility. If *try-5* is required for sperm activation, then loss of *try-5* should result in decreased fertility. To test this idea, we assayed fertility in *try-5* and *swm-1(me87) try-5* hermaphrodites and males, using the *try-5* alleles *jn2* and *tm3813*. In self-fertilizing hermaphrodites, sperm is the limiting gamete for offspring production; nearly every self sperm in a hermaphrodite will fertilize an oocyte [6], so the total self brood size is a sensitive measure of the number of functional, activated sperm produced. We found no significant difference between the number of progeny produced by *try-5* or *swm-1 try-5* mutant hermaphrodites as compared to wild-type and *swm-1* controls (Figure 3A, Figure S3A). Thus, *try-5* is not required for hermaphrodite sperm activation or fertility.

We next measured male fertility in crosses of individual males to *spe-8(hc40); dpy-4* recipient hermaphrodites. While there was a great deal of variation in the number of cross progeny produced even by wild-type males, as observed previously [25], *try-5* mutant males showed a high level of fertility and no significant difference with the wild type was observed (Figure 3B). In addition, *swm-1 try-5* males showed high levels of fertility, in some cases equivalent to that of the wild type (Figure S3B), along with suppression of the *swm-1* transfer defect (data not shown). While our assays detected no obvious fertility defects in *try-5* animals, it is possible that they might exhibit reduced fertility in other situations, *e.g.*, outside the laboratory or under conditions of sperm competition. However, these results suggest that *try-5* is not required for sperm activation or other aspects of fertility in either sex.

### *try-5* and the *spe-8* group define two pathways for sperm activation

Although *try-5* is not required for either male or hermaphrodite fertility, there is previous evidence for distinct pathways of sperm activation in males vs. hermaphrodites [12], [14], raising the possibility that the effect of *try-5* loss is masked by functional redundancy. Therefore, we tested genes in the hermaphrodite pathway for redundancy with *try-5*. The activities of a set of five genes termed the “*spe-8* group” (*spe-8*, *-12*, *-19*, *-27*, and *-29*) are required for self-sperm activation in the hermaphrodite but not for activation of male sperm (reviewed in [26]). To test whether *try-5* and the *spe-8* group function in independent, redundant activation pathways, we assayed sperm activation and male fertility in worms lacking both *try-5* and *spe-8* group activity, using the *spe-8* group mutations *spe-27(it110)* and *spe-29(it127)*. While *spe-27* mutant males are fertile and capable of generating cross progeny, we found that *spe-27; try-5* mutant males were completely infertile (Figure 3C). Similarly, while *spe-29* mutant males are fertile, *spe-29; try-5* fertility was greatly reduced as compared to the wild type (Figure 3C).

To investigate the cause of this infertility, we labeled males with MitoTracker [27] and crossed them to unlabeled recipient hermaphrodites to assay sperm transfer and migration [20]. We found that *spe-27; try-5* males were able to transfer sperm to hermaphrodites, but the transferred sperm did not migrate. Similarly, for *spe-29; try-5* males, we observed only rare instances of successful migration (Table S3 and data not shown). To determine if the migration defect was due to improper activation or a defect in migration after sperm activation, we dissected hermaphrodites immediately after their mating to *spe-27*; *try-5* males and examined transferred, MitoTracker-labeled sperm. We found that whereas *spe-27* sperm activate within fifteen minutes after transfer to a hermaphrodite, *spe-27*; *try-5* sperm fail to activate (data not shown). Thus, *spe-27; try-5* males are infertile due to failure to activate sperm upon transfer to hermaphrodites. Our findings of residual fertility and sperm migration in *spe-29; try-5* males are consistent with previous observations [17] that the single known mutation in *spe-29* leads to a weaker phenotype as compared to known null mutations in other *spe-8* group genes. These results suggest that *try-5* activity is the source of fertility in *spe-8* group mutant males; *i.e.*, the *spe-8* group and *try-5* function in two separate pathways for sperm activation, and either pathway is normally sufficient for full male fertility.

### *try-5* mutant males do not transfer activator to hermaphrodites

**Figure 4:**
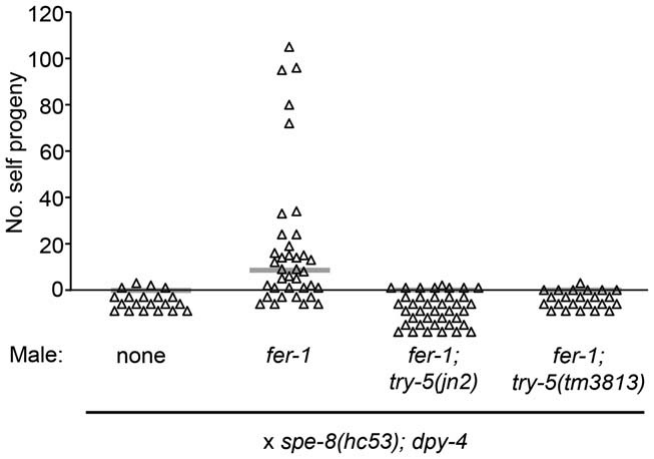
TRY-5 is required for the activation of hermaphrodite sperm by male seminal fluid. Transactivation was assayed for *try-5* males. L4 males were mated to L4 *spe-8(hc53); dpy-4* hermaphrodites in 4∶1 crosses for 48 hr, and the number of Dumpy self progeny produced during the mating period was counted. Each point represents the result from one cross. Gray bars represent medians.

To determine whether *try-5* indeed functions in the male-derived activation pathway, we used a specific assay to measure transfer of functional male activator. Wild-type male seminal fluid is capable of activating *spe-8*-group mutant hermaphrodite sperm during mating; this process is termed “transactivation” and is generally assayed using *fer-1* mutant males, which are defective for producing functional sperm, to prevent cross-progeny production [12]. We crossed either *fer-1* [25] or *fer-1; try-5* males to *spe-8(hc53); dpy-4* hermaphrodites and counted the number of self progeny generated. Crosses with *fer-1* control males resulted in transactivation approximately 58% of the time. However, *fer-1; try-5* males were rarely if ever capable of transactivating hermaphrodite sperm (Figure 4). To exclude the possibility that *fer-1; try-5* males simply might harbor a behavioral defect that reduced their mating frequency, we used MitoTracker to label males and assessed their ability to transfer sperm. We observed similar frequencies of hermaphrodites containing labeled sperm after incubation with *fer-1* males, *fer-1; try-5(jn2)* males, or *fer-1; try-5(tm3813)* males (43%, 57%, or 63%, respectively). These data indicate that *fer-1; try-5* mutants mate and transfer sperm with similar success rates as compared to the control. Thus, *try-5* mutant males are defective in transfer of the male activator responsible for transactivation of *spe-8* group hermaphrodite sperm.

### TRY-5 is expressed in and secreted from the male somatic gonad

To determine how TRY-5 functions in male sperm activation, we sought to determine where it is expressed and localized. Since we predicted that TRY-5 protein is secreted, we generated both a *Ptry-5::GFP::H2B* transcriptional reporter, a histone-H2B fusion that localizes to cell nuclei and facilitates identification of cells, and a *Ptry-5::TRY-5::GFP* translational reporter for assessing TRY-5 protein localization and function. We created stable transgenic worm strains using MosSCI (Mos1-mediated Single Copy gene Insertion [28], Tables S1 and S2) and confirmed that the *Ptry-5::TRY-5::GFP* transgene restored a premature sperm activation phenotype to *swm-1 try-5* mutants (Materials and Methods, Tables S1, S2, S4).

**Figure 5:**
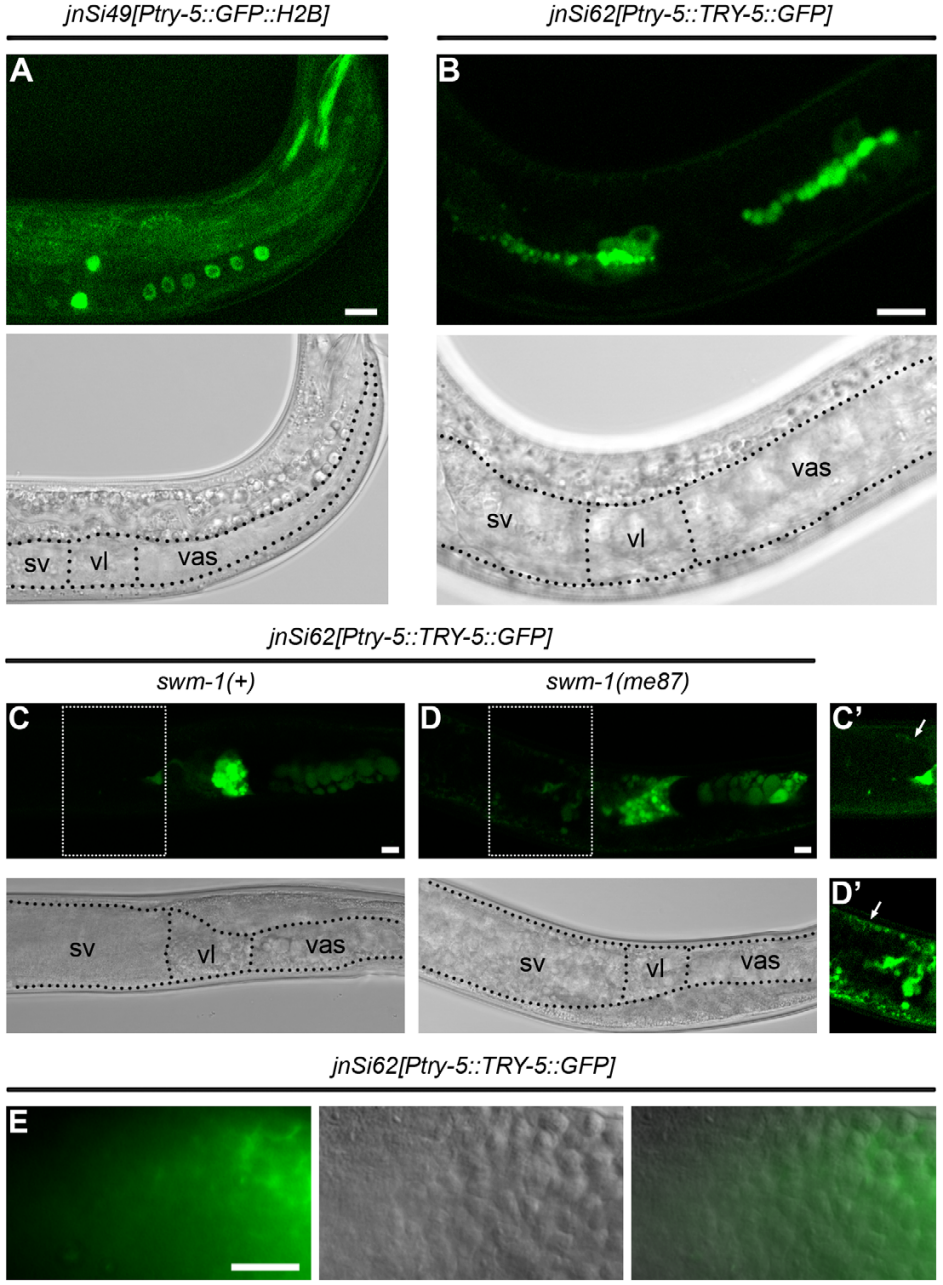
*try-5* is expressed in the male somatic gonad. Confocal and transmitted-light (TL) images of transgenic males bearing *try-5* reporter insertions. In TL images, boundaries of regions of the somatic gonad are labeled as sv, seminal vesicle; vl, valve; and vas, vas deferens. Scale bars, 10 µm. (A) *jnSi49[Ptry-5::GFP::H2B]; try-5(tm3813)* L4 male. In focal planes shown, GFP is visible in the four GFP-positive seminal vesicle cells, two of four GFP-positive valve cells, and six of twelve GFP-positive cuboidal cells. (B) *jnSi62[Ptry-5::TRY-5::GFP]; try-5(tm3813)* L4 male. TRY-5::GFP is concentrated at the apical side of seminal vesicle, valve and cuboidal cells. (C) *jnSi62[Ptry-5::TRY-5::GFP]; try-5(tm3813)* 48 hr adult male. Both large and small TRY-5::GFP globules are present in the cuboidal cells and valve region. In the seminal vesicle, TRY-5::GFP is present in the proximal region near the valve. (D) *jnSi62[Ptry-5::TRY-5::GFP]; swm-1(me87) try-5(tm3813)* 48 hr adult male. TRY-5::GFP has expanded into the seminal vesicle lumen and sperm are activated. (C′, D′) Images of seminal vesicle regions outlined in C and D with intensity levels optimized for the fainter GFP signal in these tissues. Arrows indicate GFP in the distal sheath-like cells of the seminal vesicle. (E) Paired DIC, epifluorescence and merged images of a *jnSi62[Ptry-5::TRY-5::GFP]; try-5(tm3813)* 72 hr adult male in the region of the seminal vesicle. Localization of TRY-5::GFP is correlated with activated sperm (also see Table S4 and S5).

Using the *Ptry-5::GFP::H2B* reporter, we found that the primary site of *try-5* expression was in the male somatic gonad, in particular within tissues involved in storing sperm and tissues through which sperm pass during transfer to a hermaphrodite. The *C. elegans* male gonad is essentially a long tube. At the distal end of this tube, germline stem cells reside and proliferate, and as they move proximally, they undergo meiosis and differentiate into spermatids. A subset of somatic gonadal cells surround spermatids to form a storage organ, the seminal vesicle; a more proximal set forms a channel, the vas deferens, through which sperm move during transfer. A valve structure regulates movement of sperm between the seminal vesicle and vas deferens. The vas deferens contains at least two distinct cell types, based on shape: cuboidal and elongated cells [29]. Beyond an obvious structural role, other functions of these different cell types are not known, although some of them appear to be involved in secretion [29]. Starting at the L4 larval stage, when sperm production initiates, we observed *Ptry-5::GFP::H2B* expression in several regions of the male gonad (Figure 5A): the seminal vesicle (up to seven of the twenty-three total cells in this tissue [30]), the valve region (four cells), and the twelve cuboidal cells of the vas deferens [29]. This overall pattern persisted into adulthood until at least 72 hr post L4; highest expression levels were present consistently in the valve region. We observed no expression in the hermaphrodite gonad, so gonadal expression of *try-5* is sexually dimorphic. However, we also observed low levels of expression in a few cells within the head and tail of both males and hermaphrodites (data not shown).

In worms carrying the *Ptry-5::TRY-5::GFP* reporter, the TRY-5::GFP fusion protein exhibited a localization pattern consistent with secretion from the vas deferens. Within the valve and cuboidal cells, TRY-5::GFP was localized to globular foci. In L4 larvae, most globules aligned with the apical domain that lines the developing sperm channel (Figure 5B). In mature adults, very large globules were present that tended to cluster apically, and additional small globules were present throughout the cytoplasm (Figure 5C and 5D). Such large globular structures are generally visible in adult males by DIC microscopy and diagnostic of vas deferens tissue, including within wild-type animals lacking a transgene. Based on their size and location, these large globules are likely to represent the “secretory globules” observed by electron microscopy [29].

We sometimes observed TRY-5::GFP within the lumen of the seminal vesicle, likely as a result of release from the adjacent, highly-expressing valve cells (Figure 5D and 5E, Table S5). The timing and extent of TRY-5::GFP expansion into the seminal vesicle was dependent on activity of the protease inhibitor SWM-1, the level of expression, and male age. In animals wild-type for *swm-1*, TRY-5::GFP was usually restricted to the valve cells or regions close by; when present near sperm cells, TRY-5::GFP was usually localized to a few discrete foci (data not shown). However, in animals lacking *swm-1* activity, we often observed large zones of TRY-5::GFP extending from the valve and surrounding sperm in the seminal vesicle (compare Figure 5C and 5D; see Table S5). Even in *swm-1*(+) animals, when high levels of TRY-5::GFP were present in the seminal vesicle, we almost always observed that sperm were activated (Figure 5E, Tables S4 and S5). Together, these data suggest that TRY-5 is produced by cells of the male somatic gonad and can induce sperm activation within males if it is released into the seminal vesicle. It has been observed previously that older wild-type males sometimes contain activated sperm [20], and the finding that TRY-5::GFP is released into the seminal vesicle in older males provides a basis for this phenotype. Thus, these results support a model in which TRY-5 acts locally on sperm, either to signal their activation or to generate such a signal, and SWM-1 acts to inhibit the accumulation and/or activity of TRY-5 in the seminal vesicle.

### TRY-5 is transferred to hermaphrodites during mating

**Figure 6:**
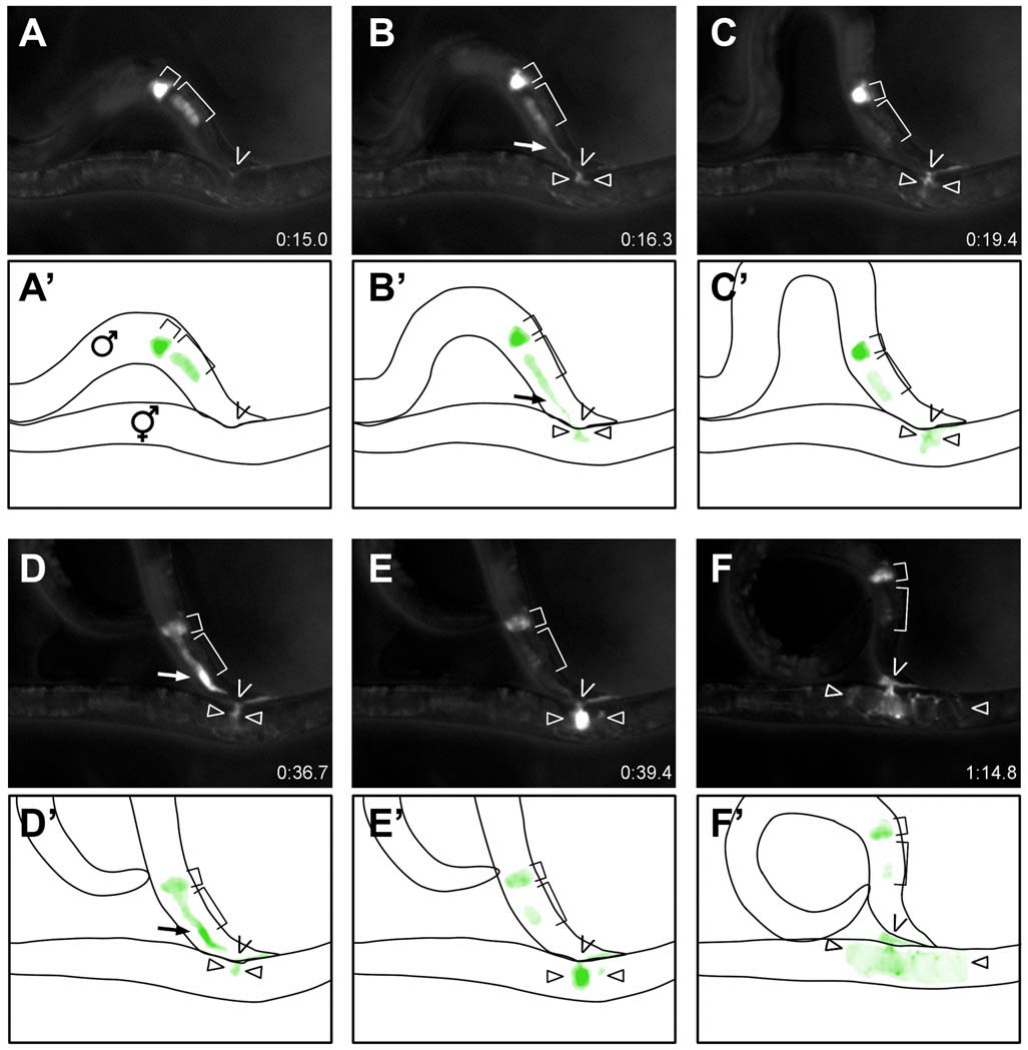
TRY-5 is transferred to hermaphrodites during mating. Selected still images (A–F) and schematics (A′–F′) depicting the time course of TRY-5::GFP transfer to an *unc-52* hermaphrodite (see Video S2). (A) TRY-5::GFP is visible within its source cells in the valve (small bracket) and vas deferens (large bracket). (B) Transfer of TRY-5::GFP from the vas deferens into the hermaphrodite. (C) Pause between transfer of the two pools. (D, E) Transfer of TRY-5::GFP from the valve into the hermaphrodite. (F) Spread of TRY-5::GFP within the uterus concomitant with transfer of sperm (data not shown). Arrows indicate TRY-5::GFP during transfer, “V” symbols indicate position of the hermaphrodite vulva, and arrowheads indicate TRY-5::GFP within the uterus. Time shown is relative to the beginning of Video S2.

Since TRY-5 localization is consistent with secretion from the male gonad, we sought to determine whether TRY-5 is transferred during mating. We placed individual MitoTracker-labeled *Ptry-5::TRY-5::GFP; try-5(tm3813)* males with *unc-52* hermaphrodites, monitored the males for mating behavior [31], and acquired fluorescence images starting at or just before spicule insertion. We observed that TRY-5::GFP was transferred to hermaphrodites during mating (Figure 6 and Video S2). Shortly after spicule insertion, TRY-5::GFP was released from the vas deferens and transferred to the hermaphrodite (Figure 6A and 6B). A brief pause without obvious transfer then occurred (Figure 6C). Next, TRY-5::GFP was released from the valve cells and travelled rapidly through the vas deferens into the hermaphrodite (Figure 6D and 6E). Movement of this valve pool was immediately followed by transfer of sperm (data not shown). After transfer, the TRY-5::GFP signal dispersed throughout the uterus (Figure 6F) and remained visible near the vulva for several minutes, if eggs were not laid immediately. This stereotypical series of events occurred for all cases (n = 5) in which the entire process was observed from spicule insertion to sperm transfer. We also observed a partial time course of five other matings, all of which were consistent with this sequence of events.

To confirm that this behavioral sequence is not unique to this specific hermaphrodite genotype, we mated *Ptry-5::TRY-5::GFP; try-5(tm3813)* males to either *unc-31* (n = 4) or *him-5 unc-76* (n = 3) hermaphrodites. We were unable to observe vas deferens TRY-5::GFP transfer in these cases due to excess hermaphrodite movement. However, we did observe that valve TRY-5::GFP transfer initiated approximately 15–55 sec after spicule insertion, which is similar to the time observed for mating with *unc-52* hermaphrodites (Figure 6, Video S2 and data not shown) and consistent with the reported timing for sperm transfer from 14.4 to 90.2 sec after spicule insertion as determined by Schindelman [32]. In summary, our data suggest that TRY-5 is a seminal fluid protein that is transferred to the hermaphrodite during copulation. Furthermore, our observations indicate that seminal fluid is released in discrete pools from specific tissues of the male gonad and that these events occur largely prior to and coincident with transfer of sperm.

## Discussion

### Activation of *C. elegans* sperm motility by a protease signal

**Figure 7:**
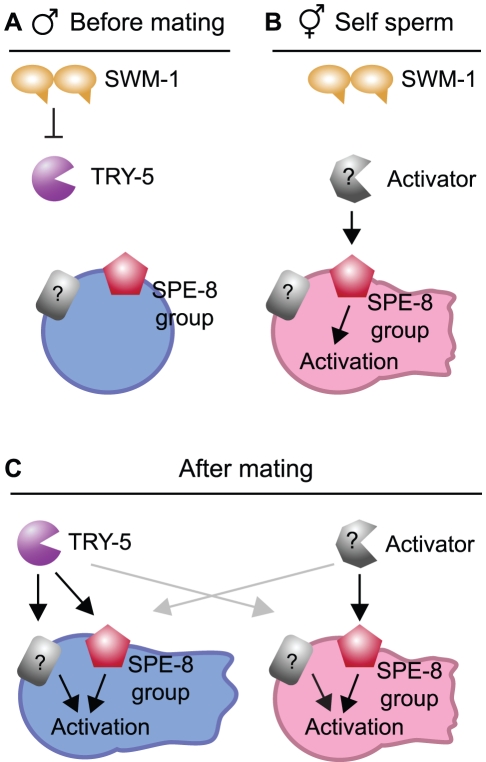
Models for the protease regulation of sperm activation in males and hermaphrodites. (A) Model: Male pathway for sperm activation, shown prior to mating when SWM-1 inhibits TRY-5 activity within the male gonad to prevent premature activation. In the absence of SWM-1, activation is signaled through interaction between TRY-5 and spermatids, likely through cleaving a target(s) on the cell surface (black arrows). Potential targets of TRY-5 include both members of the SPE-8 group and one or more additional sperm surface proteins. (B) Model: Hermaphrodite pathway for self-sperm activation. Activation is signaled through interaction between hermaphrodite activator and spermatids in the spermathecae (black arrow). This process is dependent on activity of the SPE-8 group. SWM-1 weakly antagonizes self sperm activation, suggesting that the hermaphrodite activator may be a protease. (C) Model: Pathways for activation after mating and seminal fluid transfer. When mating occurs, components of male and hermaphrodite pathways are both present and can promote sperm activation. Either pathway can activate both male and hermaphrodite sperm (grey arrows). However, hermaphrodite activator may function solely through the SPE-8 group.

We have identified a serine protease, TRY-5, which functions in *C. elegans* male sperm activation, the process by which amoeboid sperm cells become motile and competent to fertilize an egg. Based on our analysis of the defects of *try-5* mutants and the dynamic localization of a TRY-5 reporter, we propose that TRY-5 is a sperm activating signal (Figure 7A and 7C). TRY-5 function is required for premature activation of stored sperm in males lacking the protease inhibitor SWM-1. TRY-5::GFP is expressed by the male somatic gonad within secretory cells. When observed outside these cells, localization of TRY-5::GFP protein strongly correlates with the localization of activated sperm. We have directly observed the transfer of TRY-5::GFP to hermaphrodites during copulation, and *try-5* mutant males are incapable of transferring sperm activator to hermaphrodites. Together, these data strongly support a model in which TRY-5 is a component of seminal fluid that is transferred during copulation to signal sperm activation. Coupling the exposure of sperm to TRY-5 to the timing of transfer serves to ensure that sperm motility is rapidly induced at the time of - but not before - entry into a hermaphrodite's reproductive tract, thereby promoting male fertility.

Our discovery of a seminal fluid serine protease provides a mechanistic explanation for previous results linking extracellular protease activity with sperm activation in *C. elegans* and in other nematodes. *C. elegans* sperm can be activated *in vitro* by incubation with Pronase, a protease preparation that primarily contains trypsin-like activity at the pH used for these assays [11]. In *C. elegans* males, loss of the SWM-1 protease inhibitor, which should result in increased protease activity, results in increased activation [20]. Recent studies of sex determination in *C. remanei*, a male-female species, showed that females could be transformed into sperm-producing “pseudohermaphrodites,” but their sperm were not motile; production of functional, activated sperm could be achieved through additional inhibition of the *C. remanei* orthologue of *swm-1* [33]. Finally, the somatic gonad of males from the related nematode *Ascaris suum* contains a protease activity, which can activate sperm [34], [35]. Thus, a role for protease activity in promoting sperm motility appears to be conserved among nematodes.

Here we describe a novel role for a protease as a signaling molecule for differentiation of sperm to a motile form. Why would a protease be used in this context? The onset of motility in *C. elegans* sperm, as in flagellate sperm, occurs at a stage subsequent to meiotic cell division and the compaction of the haploid genome. At this stage, *C. elegans* sperm cells no longer express new protein products [26]. Therefore, to alter their behavior they must either reorganize their cellular contents in response to their environment or take in external factors. In addition, the timing of activation must be tightly controlled: *C. elegans* sperm must become motile rapidly upon entry into the hermaphrodite to avoid being lost due to the continuous outward passage of eggs [6], but early activation of motility precludes transfer of sperm from the male [20]. A protease activator provides a mechanism to trigger irreversible changes in the sperm cell surface that is readily coupled to mixing of sperm with seminal fluid. This type of activator also provides a simple mechanism to hold activation in check: the use of specific protease inhibitors such as SWM-1. We propose that the balance of TRY-5 and SWM-1 activities controls the likelihood of activation in specific locations and times within the male and hermaphrodite (Figure 7). For example, within the male gonad, SWM-1 may directly inhibit TRY-5 activity to prevent activation, allowing for sperm transfer and maintaining male fertility (Figure 7A). It is likely that additional proteases and/or inhibitors also function in this process. *try-5* mutant hermaphrodites are fertile, suggesting that hermaphrodites have an activator that is independent of TRY-5 (Figure 7B). This activator could be a protease, though its identity is not known. Male sperm sometimes activate prematurely in *try-5* mutants, suggesting that males could contain a second activator. However, if it exists, such a secondary male activator must not be competent to activate male or hermaphrodite *spe-8* group sperm.

Genetic analysis of *swm-1* had suggested that it functions to inhibit two distinct protease activities that act in parallel to promote sperm activation within males [20]. This model was based on the result that partial loss-of-function mutations affecting each of the two TIL domains of SWM-1 partially complement one another. By this model, loss of a single protease would not be expected to block sperm activation. However, we find that all SWM-1 activity works through TRY-5 in males, suggesting that both domains of SWM-1 inhibit TRY-5. The apparently separable activities of the SWM-1 TIL domains could arise from interactions with factors other than proteases. Alternatively, these results can be reconciled by a regulatory model in which SWM-1 inhibits two distinct proteases, both of which act upstream of TRY-5. It is also possible that SWM-1 might inhibit both TRY-5 and a second, TRY-5-activating protease. Consistent with these ideas, many well-known protease pathways consist of sequential cascades of activator and effector functions (e.g., [36], [37]).

As an extracellular protease, TRY-5 likely signals activation by cleaving sperm cell surface proteins and altering their activity. Some of the targets of TRY-5 may be SPE-8 group proteins, based on the fact that TRY-5 is required for transactivation, a process dependent on having some *spe-8* group activity (sperm from hermaphrodites harboring null alleles of these genes are essentially incapable of being transactivated [16], [19]). However, *spe-8* group mutant males are fertile, suggesting that SPE-8 group proteins are not essential for activation in all contexts. Thus, other targets may not be members of the SPE-8 group. The existence of such targets is further supported by our finding of additional *swm-1* suppressors (distinct from *try-5*) that show full fertility in hermaphrodites and so do not fall into the *spe-8* phenotypic class (G.M.S., unpublished data).

Could TRY-5 be functioning in some role other than as a direct activator? Sperm from *try-5* mutant males can be activated within hermaphrodites after mating, in *spe-6* mutants, or by exogenous activators *in vitro*. Thus, other activators can bypass TRY-5, and *try-5* is not required for the cellular rearrangements that occur after activation is triggered. These data support the idea that TRY-5 functions in a regulatory step of the activation process. It is clear that TRY-5 is essential for transfer of sperm activator by *C. elegans* males and its localization correlates strongly with that of activated sperm. These data strongly suggest that if TRY-5 is not the signaling molecule *per se*, its activity is intimately associated with generation of the sperm activation signal.

### TRY-5 as a component of seminal fluid

Production and transfer of seminal fluid is an important aspect of male reproduction [2], [38], [39]. TRY-5 is one of the first seminal fluid proteins identified in *C. elegans*. Indeed, it is the first directly demonstrated to be transferred at mating, and the first with a specific role in promoting gamete function. Previously, *plg-1* was identified as a seminal fluid factor required for production of a copulatory plug [40] and shown to encode a mucin-like protein with a function in male mate guarding [41]. *plg-1* is expressed within the male somatic gonad in a subset of cells that express *try-5*; interestingly, *plg-1* is not expressed within the valve region [41], from which most TRY-5 appears to be released during mating (Video S2). Thus, as in other animals [42], [43], different regions of the *C. elegans* male gonad appear to be specialized to produce specific components of seminal fluid. Furthermore, our data reveal considerable complexity in the timing of release of seminal fluid from specific tissues during the mating behavioral program.

### Regulatory logic of sperm activation in a male-hermaphrodite species

We have found that *try-5* is functionally redundant for fertility in *C. elegans*. Although *try-5* mutant males fail to transfer activator, they are fertile; however, loss of both *try-5* and *spe-8*-group function leads to complete infertility for both hermaphrodites and males (tested here with mutations in two of the *spe-8*-group genes, *spe-27* and *spe-29*). These data can be explained by the following model: *spe-27; try-5* and *spe-29; try-5* animals (1) make sperm that do not respond to hermaphrodite activator (due to loss of *spe-8*-group function) and (2) do not produce male activator (due to loss of *try-5*). In other words, *try-5* males may be fertile due not to the presence of additional activators provided by the male, but rather due to rescue of male sperm activation by a signal within the hermaphrodite (Figure 7C).

These findings of redundancy raise the question: why does *C. elegans* have *try-5*? At least part of the answer might lie in the evolutionary history of this species, which evolved from a gonochoristic (male-female) ancestor [44], [45]. As the male activator, *try-5* may represent the ancestral mode of activating sperm. Baldi et al. [33] have shown that the transition from gonochorism to androdioecy in the related species *C. remanei* requires only two steps: making sperm and activating it. Acquisition of the ability to make sperm could be advantageous, even in the initial absence of a robust self-sperm activation mechanism, as long as it tended to increase fertility. Chance encounters with a male would potentially activate hermaphrodite self sperm, as long as hermaphrodite sperm remained capable of responding to male activator. In turn, the male may have developed mechanisms to ensure his sperm were used preferentially; indeed, *C. elegans* male sperm show strong precedence over those of the hermaphrodite [6], [46]. Eventually, the hermaphrodite might evolve her own mechanism for activating sperm. The self-sperm activator in *C. elegans* is not known, but it may be a serine protease. Indirect evidence for this idea is provided by data indicating that the inhibitor SWM-1 functions in hermaphrodites: while animals mutant for the *spe-8* class gene *spe-29* have very low levels of self sperm activation and fertility, this phenotype is partially suppressed by mutations in *swm-1* [20]. However, this protease is likely distinct from TRY-5, since we have found that *try-5* is not required for either normal hermaphrodite fertility or increased activation in *spe-29; swm-1* hermaphrodites (Figure 3A, Figure S4).

Alternatively, production of TRY-5 would be advantageous for males if it is a more efficient activator than that of hermaphrodites. While our fertility assays revealed no difference between fertility of wild-type and *try-5* males, those assays were performed under highly permissive conditions: young adult animals were provided with many opportunities for mating to occur under conditions of unlimited food resources. TRY-5 might be important to increase reproductive fitness in less-than-ideal conditions. For example, activation by TRY-5 might occur more rapidly than that mediated by the hermaphrodite activator. If so, its transfer would decrease the chance that transferred sperm would be lost before they have the opportunity to migrate away from the vulva.

In summary, our work has identified a serine protease in *C. elegans* male seminal fluid that regulates the timing of sperm activation to promote male fertility. TRY-5 is transferred along with sperm during mating to couple sperm motility to entry into the hermaphrodite reproductive tract. While TRY-5 appears to be necessary for males to signal activation, hermaphrodites contain their own activator. Interestingly, these redundant pathways are competent to activate sperm from either sex, providing insight into the strategies used by *C. elegans* to adopt a male-hermaphrodite mode of reproduction. Further dissection of these signaling pathways will require identifying targets of TRY-5 and determining the nature of the hermaphrodite activator.

## Materials and Methods

### *C. elegans* genetics

*C. elegans* strains were grown as described by Brenner [47] at 20°C, except where otherwise noted. All strains were derived from the wild-type isolate Bristol N2. To ensure a ready supply of males, a strain harboring the mutation *him-5(e1490)* [48] was used as the wild type and *him-5(e1490)* was present in all other strains unless explicitly noted. The *try-5* alleles *jn2* and *jn13* were isolated as suppressors of *swm-1(me86)* and *jn21* was isolated as a suppressor of *swm-1(me66)* (G.M.S., unpublished results). Ethyl methanesulfonate (EMS) mutagenesis was performed as in [49]. *try-5(tm3813)* was a gift of S. Mitani (National Bioresource Project, Japan). Other alleles (described in Wood [49] unless otherwise noted) were: *spe-8(hc40, hc53) I*, *fer-1(hc1*ts*) I*, *ttTi5605 II* [28], *unc-52(e444) II*, *dpy-18(e364) III*, *spe-6(hc163) III* [18], *unc-119(ed3, ed9) III* [50], *spe-27(it110) IV* [15], *spe-29(it127) IV* [17], *dpy-20(e1282) IV*, *mIs11[myo-2::GFP, pes-10::GFP, gut::GFP] IV*, *dpy-4(e1166) IV*, *unc-31(e169) IV*, *swm-1(me66, me86, me87) V* [20], *unc-76(e911) V* and *nT1[unc-?(n754) let-? qIs50 ](IV, V)*.

Strains containing mutations in both a *spe-8* group gene and *try-5* were maintained as heterozygotes using the balancer *nT1*. Homozygous *spe-8* group; *try-5* males were generated by transactivation crosses of homozygous self-sterile hermaphrodites to *swm-1* mutant males, which are competent for transferring seminal fluid but rarely transfer sperm [20]. For example, for the *spe-27 dpy-20/nT1; try-5 him-5/nT1* strain, homozygous *spe-27 dpy-20; try-5 him-5* hermaphrodites were selected and crossed to either *swm-1(me87) him-5* or *mIs11*; *swm-1(me87) him-5* males to induce production of self progeny, which can be recognized as being phenotypically Dumpy.

To screen *C. elegans* proteases for a function in sperm activation, RNAi against individual protease genes was performed on *swm-1 him-5* worm strains by feeding on agar plates essentially as described by [51]. Bacteria containing inducible RNAi clones (described in [52], [53]) were obtained from Source BioScience. Genes tested by RNAi were *try-1*, *try-2*, *try-3*, *try-5*, *try-6*, *try-7*, *try-8*, *F25E5.3*, *F25E5.4*, *F25E5.7*, and *F48E3.4*. For each gene, *swm-1(me66) him-5* and *swm-1(me86) him-5* eggs were collected on RNAi plates and allowed to grow to the L4 stage; L4 males were then transferred to a fresh RNAi plate and scored either 24 hr or 48 hr later for sperm activation.

### Microscopy

Sperm activation was assayed in virgin males collected as L4 larvae and incubated at 20°C for 48 hr, unless otherwise indicated. To examine individual sperm cells, males were dissected in sperm medium (SM) (5 mM HEPES sodium salt pH 7.4, 50 mM NaCl, 25 mM KCl, 5 mM CaCl_2_, 1 mM MgSO_4_) supplemented with 10 mM dextrose [10]. Samples were observed using differential interference contrast (DIC) microscopy and sperm were scored based on cell shape as either non-activated, if spherical, or activated, based on the presence of a pseudopod. Samples were observed using an AxioImager M1 equipped with an AxioCam MRm (Zeiss). Confocal imaging was performed using a TCS SP2 (Leica). Images were processed using ImageJ [54] and Photoshop (Adobe Systems).

### Fertility assays

Hermaphrodite self fertility was measured by picking individual hermaphrodites, transferring them to fresh plates every 1–2 days until no more eggs were laid, and counting the total progeny after worms reached the L4 stage. Cases in which hermaphrodites failed to lay oocytes or died less than four days after adulthood were excluded from analysis.

Male fertility was measured in 1∶1 crosses to *spe-8(hc40); dpy-4* hermaphrodites. L4 stage animals were placed together for 48 hr; hermaphrodites were then transferred to fresh plates without males and transferred again every 1–2 days until no more eggs were laid. All cross progeny, identifiable by their non-Dumpy phenotype, were counted after worms reached the L4 stage. Use of the *spe-8* mutation in recipient hermaphrodites allows for detection of mating even in cases where functional sperm are not transferred, since transfer of seminal fluid leads to production of self progeny [14], [20]. Cases in which mating was not confirmed or the hermaphrodite died less than three days after adulthood were excluded from analysis.

For all fertility assays, wild-type broods were measured in parallel to those of the strain being assayed to control for variations in temperature, media quality and other factors that can affect progeny production or mating efficiency.

### Sperm and seminal fluid transfer assays

To assay sperm transfer and migration, males were labeled with 1 µg/mL MitoTracker CMXRos (Invitrogen) as described by Chen et al. [27] and observed as described previously [20].

Seminal fluid transfer (transactivation, [12]) was assayed using males harboring the *fer-1(hc1*ts) mutation, which results in non-functional sperm at the restrictive temperature of 25°C [25]. L4 males were crossed in a 4∶1 ratio to L4 *spe-8(hc53); dpy-4* hermaphrodites for 48 hr at 25°C. The number of self progeny (Dumpy offspring) produced during the mating period was determined after three additional days. Any crosses resulting in cross progeny (non-Dumpy offspring) were excluded from analysis. All other crosses with recipient worms surviving to the end of the mating period were included, because no marker for successful mating is available for this assay. To assess mating frequency in different *fer-1* mutant strains, males were labeled with MitoTracker and incubated with hermaphrodites in 1∶1 crosses. Hermaphrodites were then examined after 5 hr for the presence of labeled sperm. This assay likely underestimates the total mating frequency in transactivation assays, since 1) *fer-1* sperm can not migrate and are only retained within hermaphrodites for a short time period, and 2) a higher ratio of 4 males:1 hermaphrodite was used for transactivation assays.

### Assays of *in vitro* sperm activation

Activation assays were performed essentially as in [12]. Adult virgin males were dissected to release sperm in a drop of SM on a glass slide; a chamber was formed over the cells using a coverslip supported by a thin layer of Vaseline; additional SM either with activator (200 µg/mL Pronase or 60 mM TEA) or without it (control) was wicked through this chamber; and the coverslip was completely sealed with Vaseline. An image was obtained immediately upon wicking through activator and subsequent images were obtained every 5 min for at least 25 min. Activation was scored at each time point based on cell shape. To obtain time-lapse videos, activation assays were performed as described except that images were obtained once per minute.

### Observation of TRY-5::GFP transfer

For each trial, one to two 24 hr post-L4 *Ptry-5::TRY-5::GFP; try-5(tm3813) him-5* males were placed at the center of a circle of ten *unc-52*, *unc-31* or *unc-76 him-5* virgin adult hermaphrodites. Males were observed for 10 min under transmitted light using a Leica MZ16FL microscope. Prior to or shortly after spicule insertion occurred, the light source was switched to epifluorescence and images were collected at maximum speed (an exposure time of approximately 300 msec) using an AxioCam MRm (Zeiss) until spicules were removed. If copulation was not attempted within 10 min, males were removed and replaced with fresh males.

### Molecular biology

Standard molecular biology protocols were used [55]. RNA was extracted from mixed-stage *him-5* worms using TRIzol (Invitrogen). Reactions for 5′- and 3′-RACE (rapid amplification of cDNA ends) were performed using GeneRacer (Invitrogen). The MultiSite Gateway Three-fragment Vector Construction Kit (Invitrogen) with pCFJ150 as the destination vector [28] was used to generate MosSCI donor constructs (Tables S1 and S2). Plasmid pCM1.35 was a gift from G. Seydoux [56]. For TRY-5::GFP, fusion PCR was performed as in [57]. Details of Gateway plasmid construction are listed in Table S1 and Table S2. To generate pJRS17, the 279 bp KpnI-XhoI fragment from pPD95.85 was ligated into the 4855 bp KpnI-XhoI fragment from pJRS11, thereby replacing the Ser65Cys variation present in GFP derived from pPD95.75 with the Ser65Thr variation from pPD95.85.

### Transgenic strains

To generate transgenic strains harboring extrachromosomal arrays, constructs were injected [58] into the strain *unc-119; swm-1(me86) try-5(jn2) him-5* and transgenic lines were selected based on rescue of the Unc-119 phenotype [28], [50]. Single-copy insertion (MosSCI) strains were generated by the direct insertion technique into the Mos1 insertion site *ttTi5605* as described by Frokjaer-Jensen [28]. Targeting constructs were coinjected with *Pglh-2::transposase* as the source of Mos transposase and coinjection markers labeling pharyngeal muscle (*Pmyo-2::mCherry*), body wall muscle (*Pmyo-3::mCherry*), and neurons (*Prab-3::mCherry*) [28].

## Supporting Information

Figure S1 High-resolution images of adult males showing suppression of *swm-1(me87)* premature sperm activation by *try-5(tm3813)*. (A and A′) *swm-1(me87)* male from Figure 1B. Prematurely activated sperm within the seminal vesicle result in a disorganized appearance. Arrows indicate a subset of individual spermatozoa for which pseudopods are visible. (B and B′) *swm-1(me87) try-5(tm3813)* male from Figure 1C. Non-activated spermatids, containing condensed nuclei and distinctive grainy cytoplasm, are present throughout the seminal vesicle. Individual cell boundaries are often not visible by DIC; to convey packing together of these cells, arrowheads indicate the nuclei of two adjacent spermatids.
(TIF)

Figure S2 TRY-5 is a serine protease. Alignment of TRY-5 with the serine proteases trypsin, chymotrypsin and elastase (accession numbers NP_002760, NP_001897, and NP_031378). The signal sequence was predicted for TRY-5 using SignalP 3.0 [60]. Positions of *try-5* alleles are shown. Shading corresponds to identities (black) or similarities (grey) among two or more family members. Arrows indicate residues of the active site. Arrowheads indicate residues important for substrate binding.
(TIF)

Figure S3 *swm-1 try-5* double mutant hermaphrodites and males are fertile. Assays of hermaphrodite self fertility and male fertility. (A) *swm-1 try-5* double mutant hermaphrodites have wild-type fertility levels. Columns indicate average brood size of self-fertilizing hermaphrodites. Error bars represent standard error of the mean. (B) *swm-1 try-5* males have improved fertility as compared to *swm-1* males. Although the fertility of double mutants was always significantly higher than that of *swm-1*, variable levels of suppression were observed for the *swm-1 try-5(tm3813)* strain. The results of two representative experiments are shown. Each point represents the result of an individual cross; gray lines represent medians. For Repeat 1, *swm-1 try-5(tm3813)* fertility did not differ from that of wild-type males (p = 0.67, Mann-Whitney U Test), a result obtained twice. For Repeat 2, *swm-1 try-5(tm3813)* fertility did differ from that of wild-type males (p = 0.003, Mann-Whitney U Test), a result that was also obtained twice. For both repeats, *swm-1 try-5(jn2)* fertility did not differ from that of wild-type males (Repeat 1: p = 0.97, Repeat 2: p = 0.12; Mann-Whitney U Test).
(TIF)

Figure S4 *try-5* activity is not required in hermaphrodites for suppression of *spe-29* sterility by *swm-1*. Assay of hermaphrodite self fertility. Total self-progeny broods from individual hermaphrodites were counted for each strain (Text S1). Each point represents the total self progeny from an individual hermaphrodite; lines indicate the median for each set. Three replicates of the experiment were performed, with equivalent results; data from one such replicate are shown. *spe-29; swm-1* and *spe-29; swm-1 try-5* hermaphrodite fertility were each significantly different when compared to *spe-29* fertility (p<10^−6^, Mann-Whitney U test). Their fertility was not significantly different when compared to each other (p = 0.65). In addition to the listed genotypes, all strains also contained the mutation *dpy-20(e1282)*.
(TIF)

Table S1 Primers used for construction of Gateway Donor plasmids.
(DOC)

Table S2 Donor plasmids used for construction of destination constructs.
(DOC)

Table S3 *spe-8* group; *try-5* sperm do not migrate after transfer to a hermaphrodite.
(DOC)

Table S4 Premature sperm activation depends on TRY-5 and SWM-1 and increases with male age.
(DOC)

Table S5 Correlation between sperm activation and TRY-5::GFP localization.
(DOC)

Text S1 Quantification of *spe-29*.
(DOC)

Video S1 *try-5* sperm activate when treated with Pronase. *try-5(tm3813)* spermatids were treated with 200 µg/ml Pronase and images were acquired once a minute for 30 min. After activating, some sperm cells extend their pseudopods and crawl across the slide. Selected time points are shown in Figure 2B and 2C.
(MOV)

Video S2 TRY-5::GFP is transferred during mating. Transfer of TRY-5::GFP from a *Ptry-5::TRY-5::GFP; try-5(tm3813) him-5* male to an *unc-52* hermaphrodite. Details are described in Results and selected time points are shown in Figure 6.
(MOV)
