# Supplementary figures and images for: TRY-5 Is a Sperm-Activating Protease in Caenorhabditis elegans Seminal Fluid
Source: PLoS Genet. 2011 Nov 17;7(11):e1002375. doi: 10.1371/journal.pgen.1002375 (PMC3219595; doi:10.1371/journal.pgen.1002375)

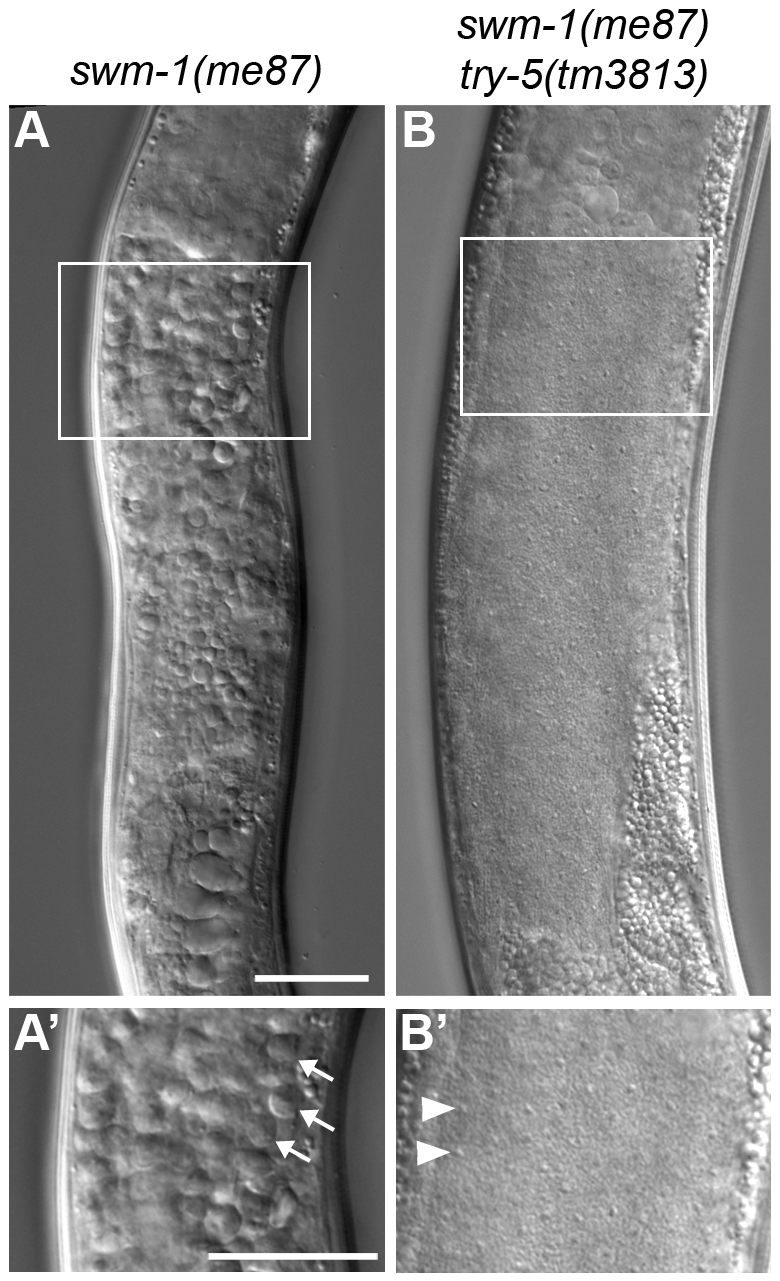

Supplement: Figure S1 — High-resolution images of adult males showing suppression of swm-1(me87) premature sperm activation by try-5(tm3813). (A and A′) swm-1(me87) male from Figure 1B. Prematurely activated sperm within the seminal vesicle result in a disorganized appearance. Arrows indicate a subset of individual spermatozoa for which pseudopods are visible. (B and B′) swm-1(me87) try-5(tm3813) male from Figure 1C. Non-activated spermatids, containing condensed nuclei and distinctive grainy cytoplasm, are present throughout the seminal vesicle. Individual cell boundaries are often not visible by DIC; to convey packing together of these cells, arrowheads indicate the nuclei of two adjacent spermatids. (TIF) [file pgen.1002375.s001.tif]

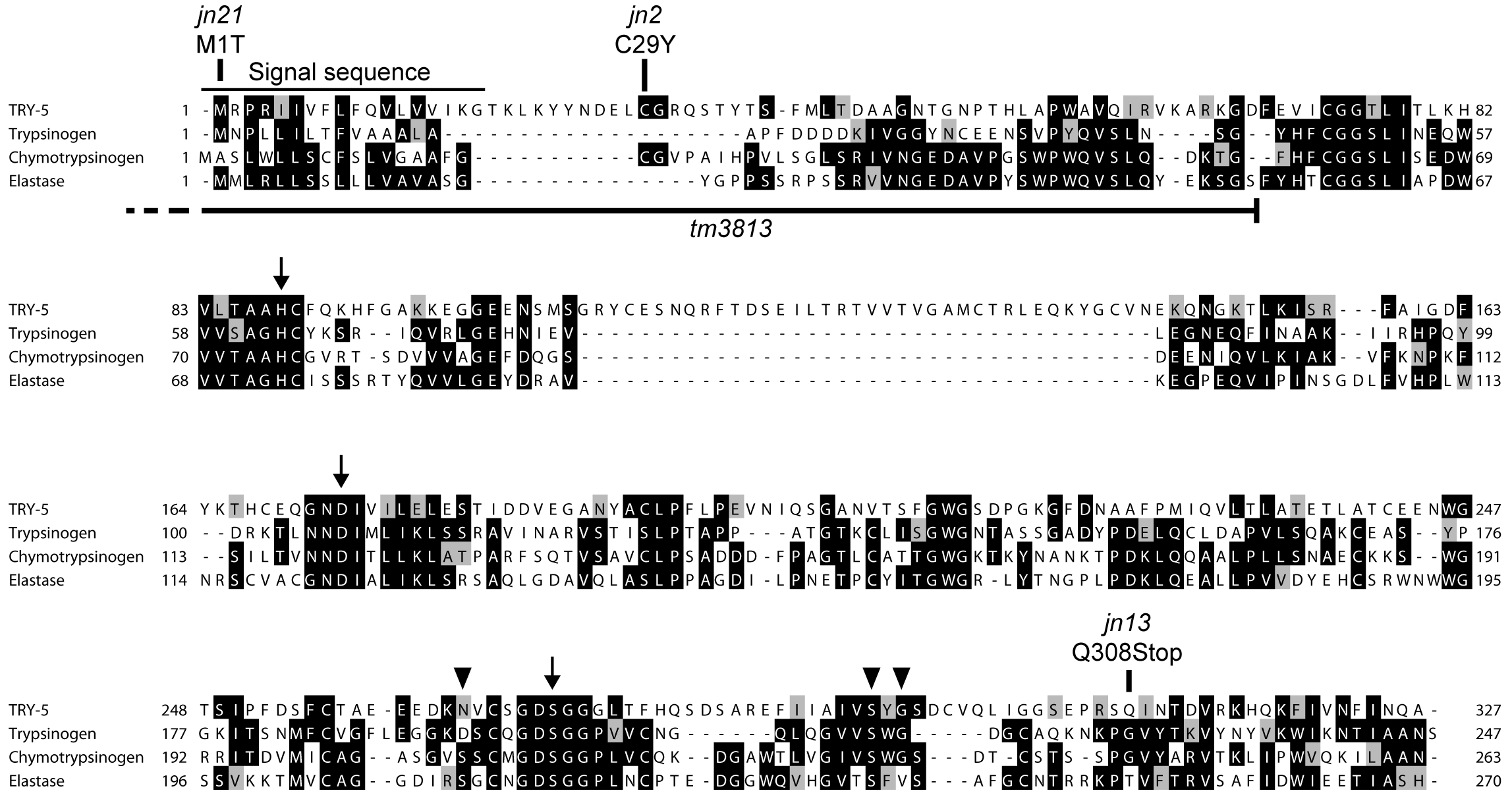

Supplement: Figure S2 — TRY-5 is a serine protease. Alignment of TRY-5 with the serine proteases trypsin, chymotrypsin and elastase (accession numbers NP_002760, NP_001897, and NP_031378). The signal sequence was predicted for TRY-5 using SignalP 3.0 [60]. Positions of try-5 alleles are shown. Shading corresponds to identities (black) or similarities (grey) among two or more family members. Arrows indicate residues of the active site. Arrowheads indicate residues important for substrate binding. (TIF) [file pgen.1002375.s002.tif]

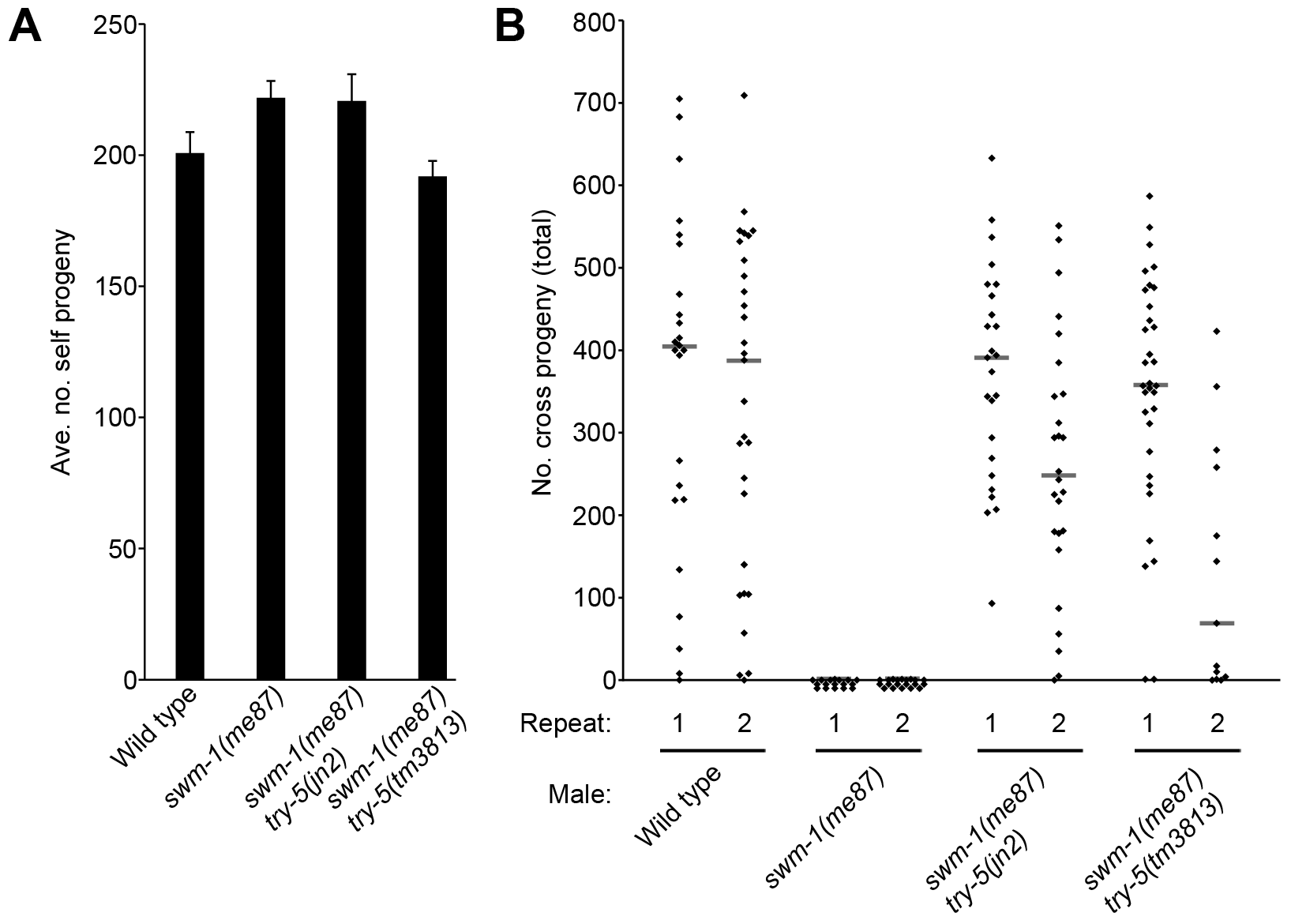

Supplement: Figure S3 — swm-1 try-5 double mutant hermaphrodites and males are fertile. Assays of hermaphrodite self fertility and male fertility. (A) swm-1 try-5 double mutant hermaphrodites have wild-type fertility levels. Columns indicate average brood size of self-fertilizing hermaphrodites. Error bars represent standard error of the mean. (B) swm-1 try-5 males have improved fertility as compared to swm-1 males. Although the fertility of double mutants was always significantly higher than that of swm-1, variable levels of suppression were observed for the swm-1 try-5(tm3813) strain. The results of two representative experiments are shown. Each point represents the result of an individual cross; gray lines represent medians. For Repeat 1, swm-1 try-5(tm3813) fertility did not differ from that of wild-type males (p = 0.67, Mann-Whitney U Test), a result obtained twice. For Repeat 2, swm-1 try-5(tm3813) fertility did differ from that of wild-type males (p = 0.003, Mann-Whitney U Test), a result that was also obtained twice. For both repeats, swm-1 try-5(jn2) fertility did not differ from that of wild-type males (Repeat 1: p = 0.97, Repeat 2: p = 0.12; Mann-Whitney U Test). (TIF) [file pgen.1002375.s003.tif]

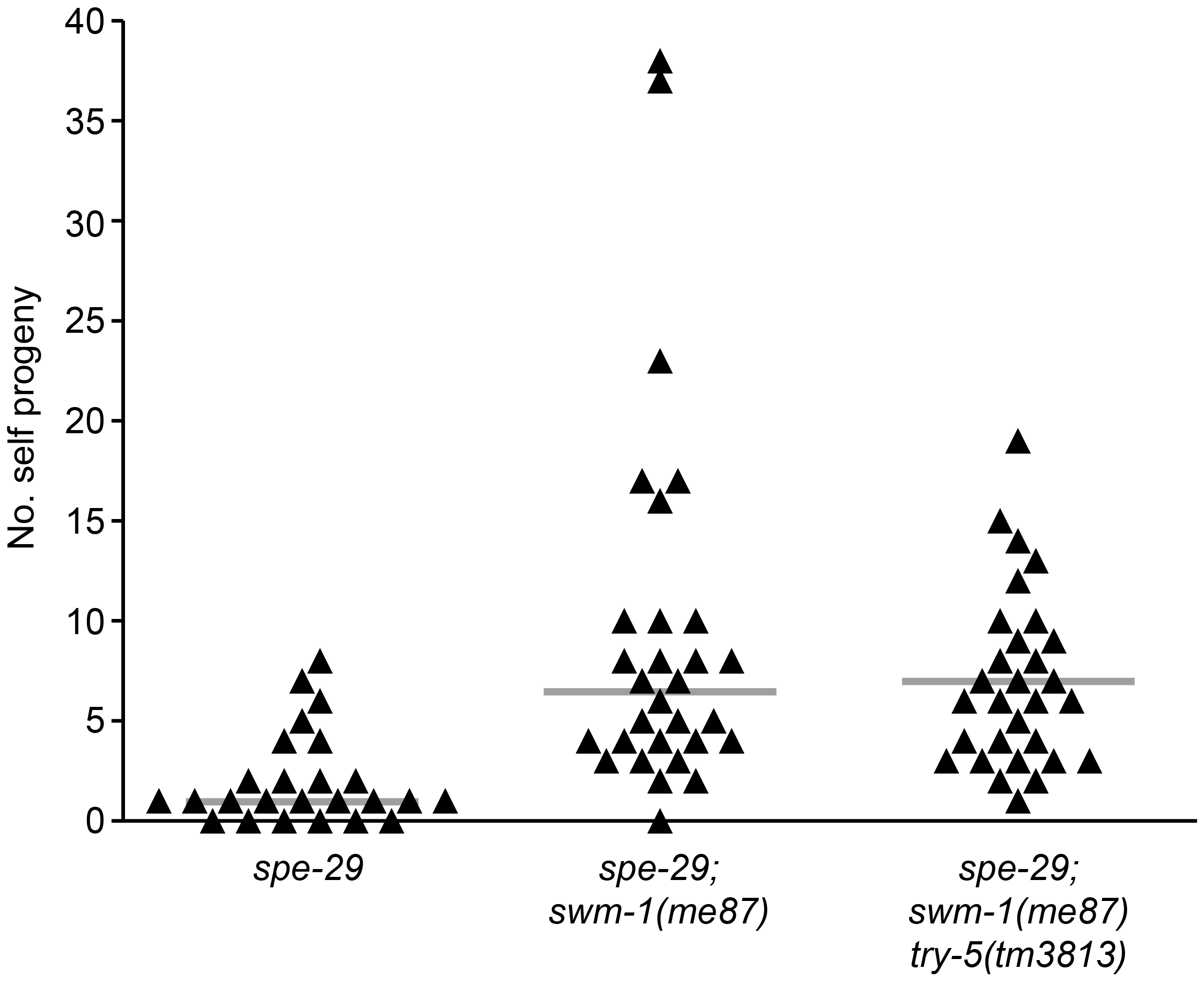

Supplement: Figure S4 — try-5 activity is not required in hermaphrodites for suppression of spe-29 sterility by swm-1. Assay of hermaphrodite self fertility. Total self-progeny broods from individual hermaphrodites were counted for each strain (Text S1). Each point represents the total self progeny from an individual hermaphrodite; lines indicate the median for each set. Three replicates of the experiment were performed, with equivalent results; data from one such replicate are shown. spe-29; swm-1 and spe-29; swm-1 try-5 hermaphrodite fertility were each significantly different when compared to spe-29 fertility (p<10−6, Mann-Whitney U test). Their fertility was not significantly different when compared to each other (p = 0.65). In addition to the listed genotypes, all strains also contained the mutation dpy-20(e1282). (TIF) [file pgen.1002375.s004.tif]
