# Supplementary material for: TRY-5 Is a Sperm-Activating Protease in Caenorhabditis elegans Seminal Fluid
Source: PLoS Genet. 2011 Nov 17;7(11):e1002375. doi: 10.1371/journal.pgen.1002375 (PMC3219595; doi:10.1371/journal.pgen.1002375)
Supplement: Table S1 — Primers used for construction of Gateway Donor plasmids. (DOC) [file pgen.1002375.s005.doc]

**Table S1. Primers used for construction of Gateway Donor plasmids.**

| **Plasmid** | **Fragment description** | **Length** | **Gateway vector** | **Forward primer^1^** | **Reverse primer^1^** |
| --- | --- | --- | --- | --- | --- |
| pJRS13 | *try-5* promoter | 1857 bp | pDONR P4-P1r | ggggacaactttgtatagaaaagttgtgcttgtcctcacactgctc | ggggactgcttttttgtacaaacttgttgaatttgaattcccgctg |
| pJRS8 | *try-5* 3’ UTR | 776 bp | pDONR P2r-P3 | ggggacagctttcttgtacaaagtggcttgattctttgttcacattcaa | ggggacaactttgtataataaagttgagctgagcattttgggagtctgactc |
| pJRS7 | *try-5* coding region | 1589 bp | pDONR 221 | ggggacaagtttgtacaaaaaagcaggctatgcgtccccgaataattgtattcct | ggggaccactttgtacaagaaagctgggtaagcttgattaataaaattcacg |
| pJRS11^2^ | *try-5::GFP(S65C)* | 2590 bp | pDONR 221 | ggggacaagtttgtacaaaaaagcaggctatgcgtccccgaataattgtattcct | ggggaccactttgtacaagaaagctgggtctatttgtatagttcatccatgcc |
| pJRS11 | *try-5* | 1631 bp | NA^3^ | agcgggaattcaaattcaaatgcgt | AGTCGACCTGCAGGCATGCAAGCTAGCTTGATTAATAAAATTCACG |
| pJRS11 | GFP(S65C)^4^ | 1892 bp | NA | AGCTTGCATGCCTGCAGGTCG | AAGGGCCCGTACGGCCGACTA |

^1^ For primers, lower-case type indicates spacer and *att* site sequences; upper-case type indicates gene-specific sequences. Genomic *him-5* DNA was used as a template unless noted.

^2^ To generate the *try-5::GFP(S65C)* construct pJRS11, fusion PCR was performed using the 1631bp *try-5* and 1892bp *GFP* fragments listed.

^3^ NA, not applicable.

^4^ The plasmid pPD95.75 (gift of A. Fire) was used as a template.
