# Supplementary material for: TRY-5 Is a Sperm-Activating Protease in Caenorhabditis elegans Seminal Fluid
Source: PLoS Genet. 2011 Nov 17;7(11):e1002375. doi: 10.1371/journal.pgen.1002375 (PMC3219595; doi:10.1371/journal.pgen.1002375)
Supplement: Table S2 — Donor plasmids used for construction of destination constructs. (DOC) [file pgen.1002375.s006.doc]

**Table S2. Donor plasmids used for construction of destination constructs.**

| **Plasmid** | **Description** | **Position 1** | **Position 2** | **Position 3** | **Vector** |
| --- | --- | --- | --- | --- | --- |
| pJRS14 | *Ptry-5::try-5::try-5* 3’ UTR | pJRS13 | pJRS7 | pJRS8 | pCFJ150 |
| pJRS18 | *Ptry-5::try-5::GFP(S65T)::try-5* 3’ UTR | pJRS13 | pJRS17 | pJRS8 | pCFJ150 |
| pJRS22 | *Ptry-5::GFP::H2B::try-5* 3’ UTR | pJRS13 | pCM1.35 | pJRS8 | pCFJ150 |
