# Supplementary material for: TRY-5 Is a Sperm-Activating Protease in Caenorhabditis elegans Seminal Fluid
Source: PLoS Genet. 2011 Nov 17;7(11):e1002375. doi: 10.1371/journal.pgen.1002375 (PMC3219595; doi:10.1371/journal.pgen.1002375)
Supplement: Table S3 — spe-8 group; try-5 sperm do not migrate after transfer to a hermaphrodite. (DOC) [file pgen.1002375.s007.doc]

**Table S3. *spe-8* group*; try-5*** sperm do not migrate after transfer to a hermaphrodite.

| **Genotype** | **Total crosses** | **No. transfer**^1^ | **No. migration**^2^ |
| --- | --- | --- | --- |
| *spe-27* | 45 | 21 | 12 |
| *spe-27; try-5(tm3813)* | 42 | 19 | 0 |
| *spe-29* | 50 | 34 | 32 |
| *spe-29; try-5(tm3813)* | 50 | 35 | 1 |

^1^Number of crosses in which MitoTracker-labeled sperm were observed inside recipients.

^2^Number of crosses in which sperm migrated to the spermathecae.
