# Supplementary material for: TRY-5 Is a Sperm-Activating Protease in Caenorhabditis elegans Seminal Fluid
Source: PLoS Genet. 2011 Nov 17;7(11):e1002375. doi: 10.1371/journal.pgen.1002375 (PMC3219595; doi:10.1371/journal.pgen.1002375)
Supplement: Table S4 — Premature sperm activation depends on TRY-5 and SWM-1 and increases with male age. (DOC) [file pgen.1002375.s008.doc]

**Table S4. Premature sperm activation depends on TRY-5 and SWM-1 and increases with male age.**

| **Genotype** | **Age**^1^ | **nonAct** | **partAct** | **fullAct** | **Total** |
| --- | --- | --- | --- | --- | --- |
| Wild type | 24hr | 56 | 0 | 0 | 56 |
|  | 48hr | 47 | 0 | 0 | 47 |
|  | 72hr | 51 | 5 | 1 | 57 |
| *swm-1(me87)* | 24hr | 1 | 1 | 51 | 53 |
|  | 48hr | 0 | 0 | 49 | 49 |
|  | 72hr | 0 | 1 | 52 | 53 |
| *try-5(tm3813)* | 24hr | 53 | 0 | 0 | 53 |
|  | 48hr | 44 | 0 | 0 | 44 |
|  | 72hr | 72 | 0 | 0 | 72 |
| *swm-1(me87) try-5(tm3813)* | 24hr | 53 | 0 | 0 | 53 |
|  | 48hr | 50 | 0 | 0 | 50 |
|  | 72hr | 51 | 0 | 0 | 51 |
| *jnSi62[Ptry-5::TRY-5::GFP]; try-5(tm3813)* | 24hr | 55 | 0 | 0 | 55 |
|  | 48hr | 51 | 0 | 0 | 51 |
|  | 72hr | 120 | 4 | 0 | 124 |
| *jnSi62[Ptry-5::TRY-5::GFP]; swm-1(me87) try-5(tm3813)* | 24hr | 22 | 11 | 9 | 42 |
|  | 48hr | 0 | 0 | 54 | 54 |
|  | 72hr | 0 | 0 | 67 | 67 |

^1^L4 larval stage males were isolated from hermaphrodites and incubated at 20°C for the indicated number of hours before observation.
