# Supplementary material for: TRY-5 Is a Sperm-Activating Protease in Caenorhabditis elegans Seminal Fluid
Source: PLoS Genet. 2011 Nov 17;7(11):e1002375. doi: 10.1371/journal.pgen.1002375 (PMC3219595; doi:10.1371/journal.pgen.1002375)
Supplement: Table S5 — Correlation between sperm activation and TRY-5::GFP localization. (DOC) [file pgen.1002375.s009.doc]

**Table S5. Correlation between sperm activation and TRY-5::GFP localization.**

|  |  | **No TRY-5::GFP present within sperm zone^1^** | | | **TRY-5::GFP foci within sperm zone^2^** | | | **TRY-5::GFP around cells in sperm zone^3^** | | |  |
| --- | --- | --- | --- | --- | --- | --- | --- | --- | --- | --- | --- |
| **Genotype** | **Age^4^** | **nonAct** | **partAct** | **fullAct** | **nonAct** | **partAct** | **fullAct** | **nonAct** | **partAct** | **fullAct** | **Total** |
| *jnSi62[Ptry-5::TRY-5::*  *GFP]; try-5(tm3813)* | 24hr | 5 | 0 | 0 | 50 | 0 | 0 | 0 | 0 | 0 | 55 |
|  | 48hr | 25 | 0 | 0 | 26 | 0 | 0 | 0 | 0 | 0 | 51 |
|  | 72hr | 43 | 0 | 0 | 75 | 0 | 0 | 2 | 4 | 0 | 124 |
| *jnSi62[Ptry-5::TRY-5::*  *GFP]; swm-1(me87)*  *try-5(tm3813)* | 24hr | 12 | 0 | 0 | 10 | 2 | 0 | 0 | 9 | 9 | 42 |
|  | 48hr | 0 | 0 | 0 | 0 | 0 | 0 | 0 | 0 | 54 | 54 |
|  | 72hr | 0 | 0 | 0 | 0 | 0 | 0 | 0 | 0 | 67 | 67 |

^1^No TRY-5::GFP was visible adjacent to sperm cells in the seminal vesicle lumen.

^2^Discrete foci of TRY-5::GFP were observed adjacent to sperm cells within the seminal vesicle lumen.

^3^Areas of TRY-5::GFP were present in a dispersed honeycomb-like pattern surrounding sperm cells in the seminal vesicle lumen.

^4^L4 larval stage males were isolated from hermaphrodites and incubated at 20°C for the indicated number of hours before observation.
