# Supplementary material for: TRY-5 Is a Sperm-Activating Protease in Caenorhabditis elegans Seminal Fluid
Source: PLoS Genet. 2011 Nov 17;7(11):e1002375. doi: 10.1371/journal.pgen.1002375 (PMC3219595; doi:10.1371/journal.pgen.1002375)
Supplement: Text S1 — Quantification of spe-29. (DOC) [file pgen.1002375.s010.doc]

**Supporting Methods**

**Quantification of *spe-29* suppression**

Single L4 hermaphrodites were placed on individual plates and transferred on day 2, 4 and 6. Dumpy self progeny were counted once all worms had reached at least the L4 stage. Brood counts were performed on all strains in parallel. Cases in which hermaphrodites died prior to the sixth day were excluded. The *spe-29 dpy-20; swm-1 try-5 him-5* strain was maintained by transactivation crosses in which *swm-1* males were crossed to Dumpy self progeny in each generation.
